# Vitamin D3 alleviates inflammation in ulcerative colitis by activating the VDR-NLRP6 signaling pathway

**DOI:** 10.3389/fimmu.2023.1135930

**Published:** 2023-02-08

**Authors:** Hongliang Gao, He Zhou, Zhiqiang Zhang, Jianshu Gao, Jian Li, Xinxia Li

**Affiliations:** ^1^ Pathology Center, Xinjiang Medical University Affiliated Tumor Hospital, Urumqi, Xinjiang, China; ^2^ The Second Department of Gastroenterology, the First Affiliated Hospital of Xinjiang Medical University, Urumqi, Xinjiang, China; ^3^ State Key Laboratory of Cancer Biology, National Clinical Research Center for Digestive Diseases and Xijing Hospital of Digestive Diseases, Fourth Military Medical University, Xi’an, Shaanxi, China

**Keywords:** VDR, NLRP6 inflammasome, ulcerative colitis, VD_3_, ATAC-seq

## Abstract

Inflammation is a key factor in the development of ulcerative colitis (UC). 1,25-dihydroxyvitamin D_3_ (1,25(OH)_2_D_3_, VD_3_), as the major active ingredient of vitamin D and an anti-inflammatory activator, is closely related to the initiation and development of UC, but its regulatory mechanism remains unclear. In this study, we carried out histological and physiological analyses in UC patients and UC mice. RNA sequencing (RNA-seq), assays for transposase-accessible chromatin with high-throughput sequencing (ATAC-seq), chromatin immunoprecipitation (ChIP) assays and protein and mRNA expression were performed to analyze and identify the potential molecular mechanism in UC mice and lipopolysaccharide (LPS)-induced mouse intestinal epithelial cells (MIECs). Moreover, we established nucleotide-binding oligomerization domain (NOD)-like receptor protein *nlrp6* ^-/-^ mice and siRNA-NLRP6 MIECs to further characterize the role of NLRP6 in anti-inflammation of VD_3_. Our study revealed that VD_3_ abolished NOD-like receptor protein 6 (NLRP6) inflammasome activation, suppressing NLRP6, apoptosis-associated speck-like protein (ASC) and Caspase-1 levels *via* the vitamin D receptor (VDR). ChIP and ATAC-seq showed that VDR transcriptionally repressed NLRP6 by binding to vitamin D response elements (VDREs) in the promoter of NLRP6, impairing UC development. Importantly, VD_3_ had both preventive and therapeutic effects on the UC mouse model *via* inhibition of NLRP6 inflammasome activation. Our results demonstrated that VD_3_ substantially represses inflammation and the development of UC *in vivo*. These findings reveal a new mechanism by which VD_3_ affects inflammation in UC by regulating the expression of NLRP6 and show the potential clinical use of VD_3_ in autoimmune syndromes or other NLRP6 inflammasome-driven inflammatory diseases.

## Introduction

UC is a kind of local recurrence of intestinal inflammatory disease because of the complexity and long course of the disease and its wide range of lesions and is recognized as a cancer-plus lesion of colon cancer (1). At present, hormones and amino salicylic acid drugs are still used as the first-choice drugs in treatment, but the long-term use of such drugs has large adverse reactions (2). Therefore, it has become a research topic to seek ideal drugs with weak adverse reactions that are effective, especially for the remission period.

1,25-dihydroxyvitamin D_3_ (1,25(OH)_2_D_3_, VD_3_), as the active metabolite of vitamin D, is a modulator in immunology and can stimulate the production of transforming growth factor beta1 (TGF-β1) and interleukin (IL)-4, thereby reducing inflammation (3). Studies in IL-10 knockout mice have found that when vitamin D is deficient, the animals spontaneously develop symptoms of colitis, such as blood in the stool and wasting, accompanied by a high mortality rate (4). Administration of enough VD_3_ relieves symptoms, showing that VD_3_ is associated with the pathogenesis of UC. In fact, there is increasing evidence that deficiency of active vitamin D plays an important role in the development and severity of inflammatory bowel diseases (IBDs) (5, 6). Animal experiments have shown that lack of cytochrome P450 family 27 subfamily B1 (CYP27b1) and decreased vitamin D secretion increase both the prevalence and severity of IBD (7). VD_3_ has been shown to have important regulatory functions in many autoimmune diseases (8, 9). Studies have shown that vitamin D directly regulates the T-cell antigen receptor (TCR) (10). In naive T cells, low expression of phospholipase C-y 1 (PLC) was associated with low cellular responses. The induction of PLC-yl is dependent on vitamin D and its receptor (VDR) (10). VDR is a nucleophilic protein that is an intranuclear biological macromolecule that mediates the biological effects of VD_3_ (11). In essence, it is a ligand-dependent nuclear transcription factor (12, 13). Active vitamin D binds to VDR and transcribes its downstream genes to exert diverse biological functions (14). VDR plays an important role in maintaining the body’s calcium-phosphorus metabolism and regulating cell proliferation and differentiation (15). The VDR level has a significant effect on IBD (5, 16). Experiments have shown that VDR gene polymorphisms and serum 25(OH) vitamin D levels are closely related to UC (17). Mutated VDR genotypes increase susceptibility to UC (18). All these studies show strong evidence for the application of vitamin D as an anti-inflammatory immunomodulator in IBD. However, the molecular mechanism underlying VD_3_-VDR-mediated alleviation of UC is still unclear.

Nucleotide-binding oligomerization domain (NOD)-like receptor protein 6 (NLRP6) is a novel member of the NOD-like receptor (NLR) protein family discovered to inhibit the innate immune response-related signaling pathway, and its encoding gene is located on human chromosome 11 (19). NLRP6 can interact with cysteine-containing aspartate specific proteinase-1 (Caspase-1) and apoptosis associated speck-like protein (ASC) containing caspase recruitment domain (CARD), forms an intracellular polyprotein complex (NLRP6 inflammasome) through the protein-protein linkage of the N-terminal pyrin domain (PYD), and finally produces interleukin (IL)-1β. IL cytokines such as IL-18 are involved in the inflammatory and immune responses (20, 21). NLRP6 is highly expressed in intestinal tissue (22). The NLRP6 inflammasome is considered an important player in maintaining intestinal homeostasis, and any perturbations of this pathway, especially NLRP6, ASC, caspase-1, or IL-18, may promote human IBD initiation or progression in some cases (23, 24).

In this study, we carried out a series of histological, physiological and molecular analyses in UC patients and UC mice and lipopolysaccharide (LPS)-induced MIECs to determine the role and regulatory mechanism of VD_3_ in UC progression. We demonstrated that VD_3_ was beneficial in the treatment of acute UC through VD_3_-VDR inhibiting NLRP6 transcription, which led to decreased NLRP6 inflammasome activity. These data provide an alternative for the diagnosis and treatment of UC. Considering this evidence, well-conducted clinical trials of vitamin D or its analogues in human UC patients are strongly indicated to further assess the potential therapeutic immunomodulatory properties of this underestimated nutrient.

## Materials and methods

### Patients

Inflammatory colorectal tissues were collected from January 2022 to June 2022 from patients first visiting the hospital for acute UC (n = 30), and the control tissues were from persons whose physical examinations were normal (n = 30). The diagnosis of UC complied with the relevant standards in the ‘Consensus Opinions on the Diagnosis and Treatment of IBD in China’. The study was approved by the Research and Ethics Committee of the First Affiliated Hospital of Xinjiang Medical University (Ethical approval number: 20211015-33). Patient consent was also obtained from all subjects before research.

### Construction of the dextran sulfate sodium induced acute colitis model

C57BL/6 mice (male, 8 weeks old) were purchased from Vital River Laboratories (Beijing, China). The mice were maintained in a 12 h light/dark cycle and allowed free access to food and water in the animal facility with a temperature-controlled environment. All animal experiments were performed according to the guidelines of the National Institutes of Health for Animal Care and Use and were approved by the Committee for Animal Research of Henan University. Acute UC was induced in mice by administering 3% DSS (MP Biomedical, Santa Ana, California, USA) into their drinking water for 7 d (Days 0 to 7). The mice were randomly assigned to the control, DSS, and DSS+VD_3_ groups, with 6 mice in each group, as per the Animal Research Reporting of *in vivo* Experiments (ARRIVE) guidelines. The control mice were given clean drinking water. Each mouse received a single injection of VD_3_ (100 *μ*g/kg) in the abdomen every 2 days (Days 0, 2, 4 and 6), and the drug treatments carried out on DSS-treated mice were blinded.

### Construction of the *nlrp6* ^-/-^ deficient mouse model

The *nlrp6* ^loxp/loxp^ mice and Villin-Cre mice were purchased from a commercial company (Cyagen, China) and were mated to produce offspring. The resulting offspring were mated with *nlrp6* ^loxp/loxp^ mice to obtain *nlrp6* ^loxp/loxp^Villin-Cre (*nlrp6^-/-^*) mice.

### Data source and differential expression analysis

The GSE155301 dataset consists of 6 microarray expression profiles and was downloaded from the Gene Expression Omnibus Comprehensive Website (GEO, http://www.ncbi.nlm.nih.gov/geo). Sample data were obtained from the colons of 3 dextran sulfate sodium-treated mice with acute UC and 3 healthy controls. The platform of the dataset was GPL21103 (Illumina HiSeq 4000 (Mus musculus)). The differentially expressed genes in the TLE and control groups were analysed using the GEO2R tool and were selected according to an at least 1.5-fold difference.

### ATAC-seq

In total, 6×10^5^ tissue-adherent MIECs were processed according to a previously published protocol (25), and 150 bp paired-end sequencing was performed on an Illumina Xten to yield an average of 97 M reads/sample.

### ChIP assay

The SimpleChip Plus Enzymatic Chromatin IP Kit (Cat# 9004, Cell Signalling, China) was used in the ChIP assay with mouse clone tissues. Immunoprecipitation was carried out with a specific VDR antibody (ab109234, Abcam) negatively controlled by nonspecific IgG (Cat# 3420, Cell Signalling, China). Twenty percent of the samples were reserved as ‘Input’. The primer sequences specific for the three VDREs are shown in **Supplemental Table 1**. The enriched DNA amounts were quantified with the results of IgG and the input DNA.

### Immunofluorescence staining

Immunofluorescence staining of mouse colon sections was performed as previously described (26). The sections were incubated with primary antibodies against VDR (ab109234, Abcam), NLRP6 (ab58705, Abcam), Caspase-1 (ab138483, Abcam) and ASC (ab175449, Abcam) at 4°C overnight, followed by an incubation with fluorescently labelled secondary antibodies. The sections were examined using a Leica confocal microscope (LEICA TCS SP5).

### Quantitative real-time PCR

The total RNA of each mouse colon tissues was extracted with TRIzol reagent (T9429, Sigma, US) according to the manufacturer’s instructions and reverse-transcribed with the PrimeScript™ RT Reagent Kit (TaKaRa, Japan). qRT–PCR was performed with SYBR Green Detection Mix (TaKaRa, Japan). The relative expression levels of genes in this study were normalized to actin expression and analyzed by the 2^-ΔΔCt^ method.

### Western blot analysis

The collected MIECs and mouse colon tissues were extracted using protein lysis buffer (Sigma-Aldrich, USA) and quantified *via* a bicinchoninic acid assay (Pierce, USA). Protein samples were then electrophoresed by 10% sodium dodecyl sulfate-polyacrylamide gel electrophoresis (SDS-PAGE) and transferred to a polyvinylidene difluoride membrane (PVDF, EMD Millipore, MA, USA), which was probed with antibodies against VDR (ab109234, Abcam), NLRP6 (ab58705, Abcam), Caspase-1 (ab138483, Abcam) and ASC (ab175449, Abcam) at a dilution of 1:1000. Blots were subsequently detected and visualized using an enhanced chemiluminescence detection kit (Millipore, Billerica, MA, USA) according to protocols provided by the manufacturer. A Bio-Rad scanning system was used to detect immunoreactive protein bands, and GAPDH (ab204276, Abcam) was used as a control.

### Intestinal permeability test

Fluorescein isothiocyanate (FITC)-labelled dextran (FD) was used to detect the intestinal permeability. After overnight fasting, the mice in each group were orally administered the permeability tracer FD (400 mg/kg) for 4 h. Blood was collected from the medial canthus, and the fluorescence intensity in serum was measured by spectrophotometer (excitation wavelength 490 nm, emission wavelength 530 nm).

### Transmission electron microscopy

The mouse colon tissues were collected and analysed as previously described (27). The ultrathin colon sections were detected with an electron microscope (HITACHI, Tokyo, Japan).

### Evaluation of disease activity index

Weigh the mice every day and observed their behaviors, stool characteristics, diarrhea degree and whether death occurred. Calculate as follows: (a) Stool consistency: 0, Normal; 2, Loose stool; 4, Watery diarrhea; (b) Blood stool: 0, Normal; 2, Slight bleeding; 4, Massive hemorrhage; (c) Weight loss: 0, None; 1, Decrease by 1%~5%; 2, Decrease by 5~10%; 3, Decrease by 11%~15%; 4, Decrease>15%. Evaluate each item of the mice, and the sum of a, b and c is the DAI score.

### Gene ontology and Kyoto encyclopedia of genes and genomes analysis

The functional enrichment of the differentially expressed genes (DEGs) was evaluated to obtain the genes associated with UC through GO and KEGG analyses. The functional terms for the GO enrichment analysis, including cellular component (CC), biological process (BP) and molecular function (MF) categories, were performed *via* the online tool Database for Annotation, Visualization, and Integrated Discovery (DAVID) (Version 6.8; https://david.ncifcrf.gov/home.jsp). KEGG analysis of DEGs was performed using KEGG Orthology-Based on KOBAS 2.0. The results of the enrichment were analysed by Fisher’s exact test, using P ≤ 0.05 as the significance threshold.

### Protein-protein association network analysis

The PPI network centred on VDR, NLRP6, ASC and Caspase-1 was constructed using the Search tool for the retrieval of interacting genes/proteins (STRING) database (Version 11.0; www.string-db.org), and a PPI score (medium confidence) ≥ 0.4 was defined as the cut-off value.

### Statistical analysis

SPSS 22.0 (IBM Corporation, USA) and GraphPad Prism 5.0 (GraphPad Inc., USA) were employed for statistical analyses. All data are presented as the means ± SD (standard deviation). Student’s *t test* or one-way ANOVA with Bonferroni’s *post-hoc* test were utilized for analysis of mRNA and protein levels between distinctive groups. A value of *P<0.05* was considered statistically significant.

## Results

### VDR expression is negatively correlated with inflammation in UC patients

**Figure 1:**
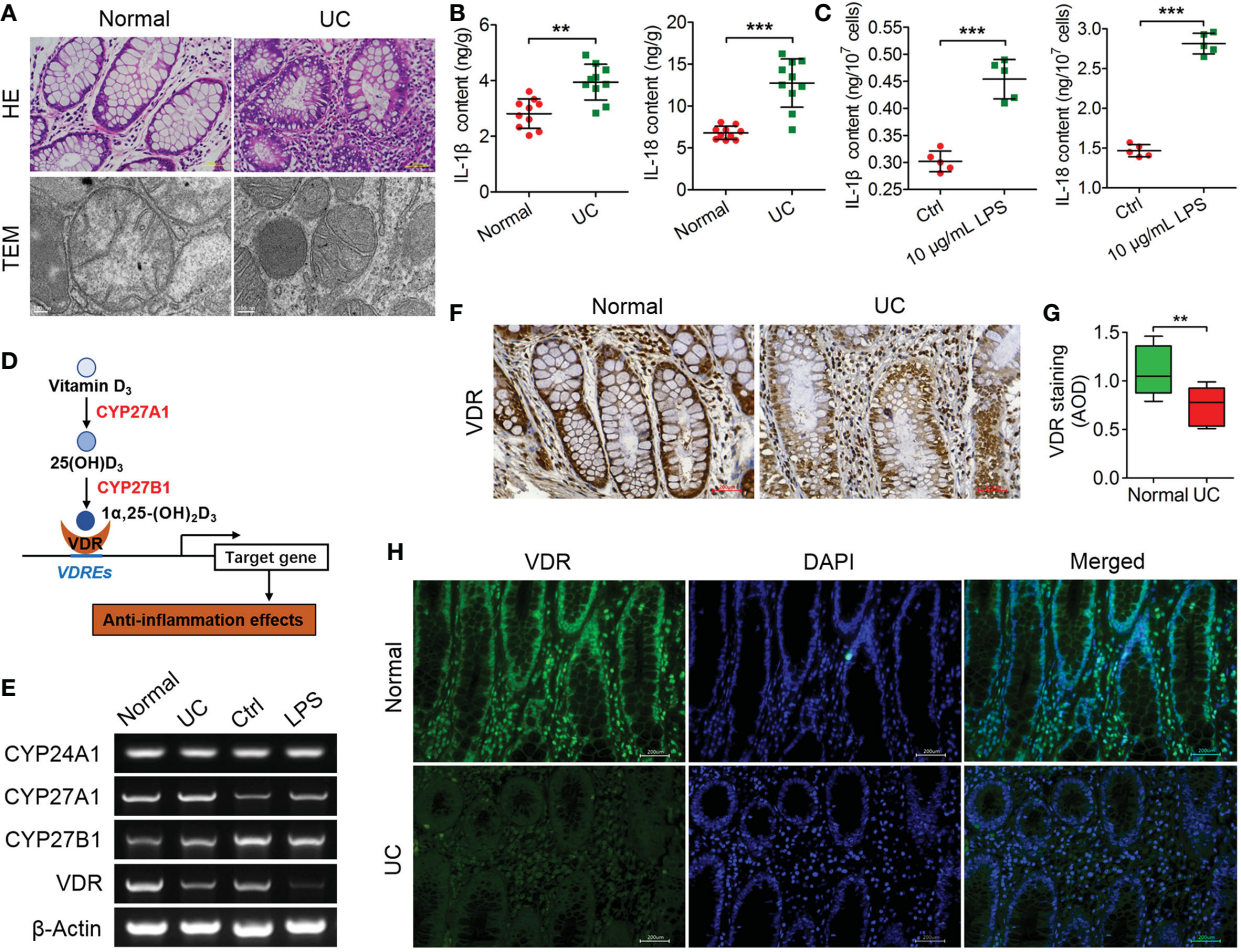
VDR is negatively correlated with inflammation in UC patients. **(A)** The pathological features of colitis are depicted by HE staining and TEM observation. **(B, C)** The contents of IL-1 and IL-18 were analysed through ELISA in UC tissues **(B)** and LPS-treated cells **(C)**. **(D)** Schematic diagram illustrating the key factors (CYP27A1, CYP27B1, VDR and CYP24A1) involved in the anabolism and catabolism of 1α,25(OH)_2_D_3_ and the regulation of target genes. **(E)** qPCR was used to analyse the mRNA expression of CYP24A1, CYP27A1, CYP27B1 and VDR in colon tissue from UC patients and LPS-treated MIECs. **(F, G)** Representative Immunohistochemical images **(F)** and quantified data **(G)** for VDR in colons from normal and representative UC patients. **(H)** The expression of VDR was measured by immunofluorescence assay in colon sections from normal and representative UC patients. The data are shown as the means ± SD; n ≥ 3, **P < 0.01, ***P <0.001.

To investigate the histopathological damage of colitis in UC patients, pathological features were analyzed through HE staining and TEM observation. The results of HE staining showed that the colonic tissue of normal individuals was integral, the mucosa had no obvious defect, the glands were neatly arranged without atrophy, and no obvious inflammatory cells infiltrated the mucosal lamina propria. However, in UC patients, the mucosal layer of the gland had atrophied or had even disappeared, replaced by a large number of inflammatory cells infiltrating the submucosa (**Figure 1A**). Furthermore, the mitochondria, endoplasmic reticulum, lysosome and nucleus in normal colon epithelial cells with regular shapes were clearly visible by TEM, and the mitochondria clearly revealed double membranes (**Figure 1A**). However, these intracellular organelles were reduced in number and blurred in UC patients (**Figure 1A**). Thus, the occurrence of UC could induce obvious pathological changes in colon tissue.

In addition, the IL-1β and IL-18 contents in UC patients were significantly increased compared to those in normal patients (**P < *0.01*, ***P < *0.001*; **Figure 1B**). The LPS (10 μg/ml)-treated MIECs confirmed the results from UC patients, as both the IL-1β and IL-18 contents were increased significantly compared to the controls (***P < *0.001*; **Figure 1C**).

VD_3_ was shown to present anti-inflammatory actions and generated the active metabolite 1α,25-(OH)_2_D_3_ mainly by the metabolizing enzymes CYP27A1, CYP27B1 and CYP24A1, which were recognized by the nuclear transcription factor VDR regulating a series of gene expressions (28, 29) (**Figure 1D**). According to the qPCR results, the mRNA expression levels of CYP24A1, CYP27A1, and CYP27B1 changed unconspicuously, but the VDR mRNA levels were decreased in UC patients and LPS-treated MIECs (**Figure 1E**). Furthermore, the immunohistochemical results showed that VDR expression was downregulated significantly in the colon tissues of UC patients (***P < *0.001*; **Figures 1F, G**). The VDR immunofluorescence signals were weak in UC patients, which confirmed the results from immunohistochemistry (**Figure 1H**).

### VD_3_ treatment attenuates pathological damage to DSS-induced UC in mice

**Figure 2:**
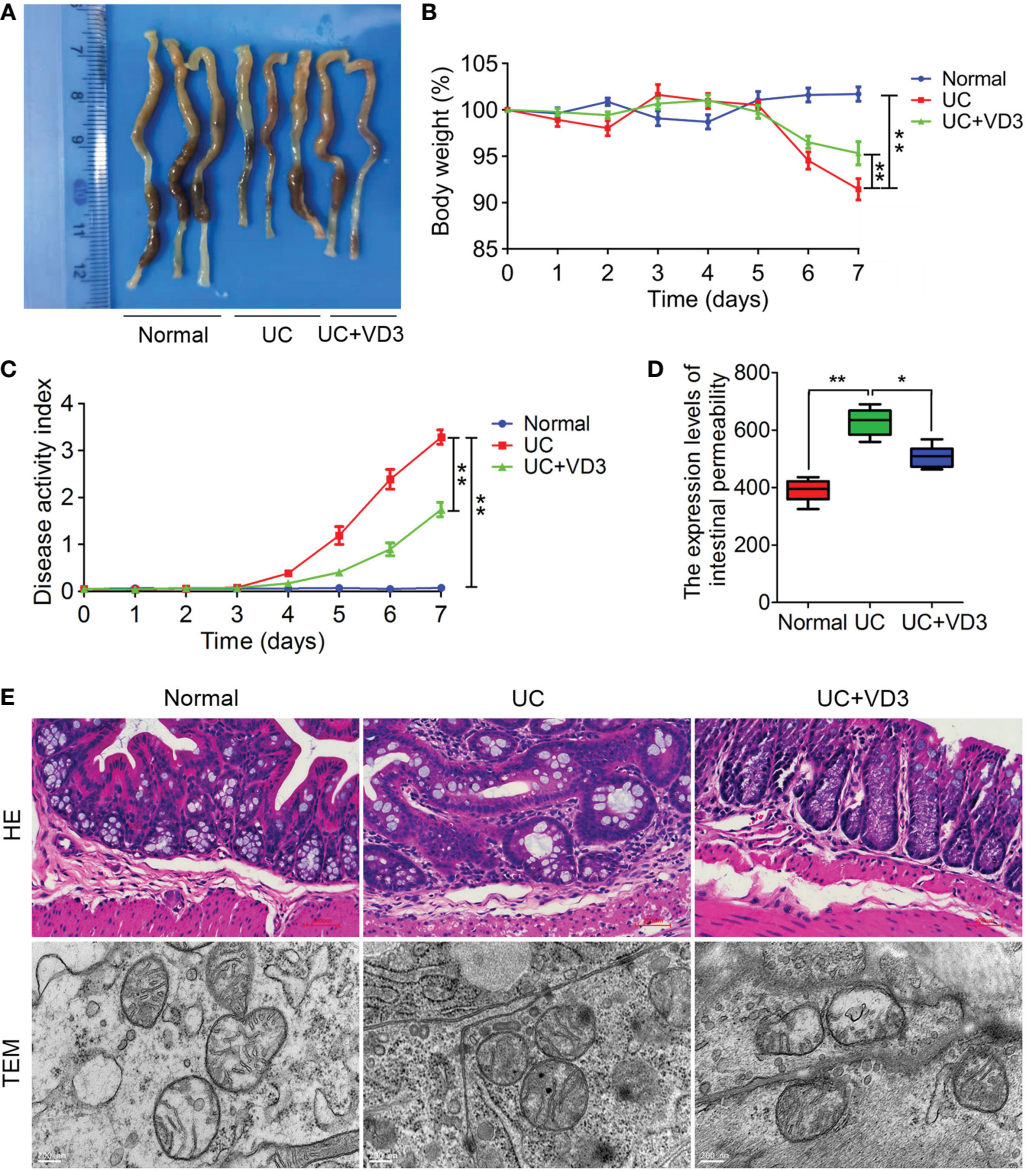
1,25(OH)_2_D_3_ alleviates DSS-induced UC in mice. **(A)** Macroscopic appearances and colon lengths of the mice were measured. **(B)** Mice were given 3% DSS in drinking water for 7 d to induce acute colitis. Body weight loss. **(C)** The DAI of these mice during the experimental period is depicted. **(D)** The intestinal permeability rate of FITC-dextran was measured. **(E)** Representative HE-stained and TEM colon sections. The data are shown as the means ± SD; n ≥ 3, *P < 0.05, **P < 0.01.

To investigate the effect of VD_3_ on UC development, mice were given 3% DSS in drinking water to induce acute colitis and administered VD_3_. Seven days later, the UC colons were shorter and smaller than the normal colons (**Figure 2A**), and the body weights of the mice were significantly reduced (**P < *0.01*; **Figure 2B**). However, VD_3_ treatment significantly mitigated the effect of UC on mouse colon tissues, and the body weight of the UC + VD_3_ group was significantly increased compared to that of the UC group (**P < *0.01*; **Figure 2B**). Moreover, the value of DAI was significantly higher in UC mice than in normal mice, but it decreased significantly after VD_3_ treatment (**P < *0.01*; **Figure 2C**). The UC mice showed a significantly high intestinal permeability rate of FD, but the UC + VD_3_ group revealed a significantly lower FD intestinal permeability rate than the UC mice (**P < *0.01*; **Figure 2D**). The UC mice exhibited damaged structure and disordered glands, massive destruction of mucosal epithelial cells, and infiltration of a large number of inflammatory cells in the submucosa, as shown by HE staining of colons, and the number of intracellular organelles in UC mice was reduced and difficult to distinguish by TEM, similar to the findings in UC patients (**Figure 2E**). However, the pathological damage after VD_3_ treatment was significantly reduced compared with that in UC mice (**Figure 2E**).

### The VDR-NLRP6 signaling pathways are involved in DSS-induced UC

**Figure 3:**
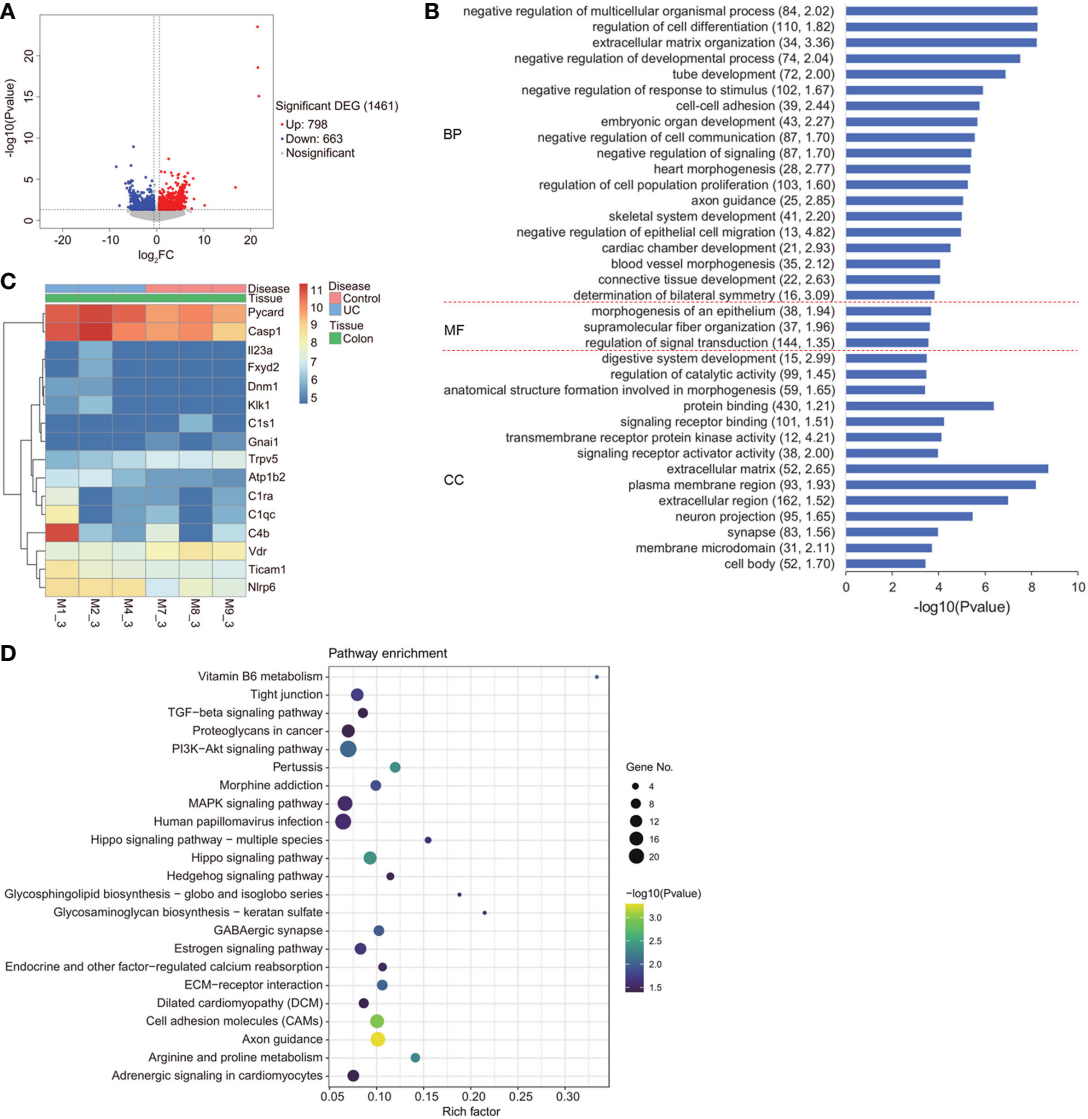
Gene expression profile of DSS-induced UC in mice. **(A)** Volcano plot of differentially expressed genes. X axis: log 2 FC; Y axis: -log10 (FDR). Red represents upregulated genes, and blue represents downregulated genes. **(B)** GO term analysis of BP, MF, and CC for the genes. The minus logarithm of the *P value* (x-axis) indicates the significance of the gene set belonging to predefined categories under the coexpression network gene background. The y-axis represents each GO category. **(C)** A heatmap depicting the gene expression profiles of vitamin B6 metabolism and the pertussis signalling pathway in the colon of the DSS mouse model and healthy controls. X axis: sample name; Y axis: gene name. **(D)** Enriched upregulated and downregulated genes, as determined by KEGG pathway analysis.

To determine the molecular mechanism underlying the effects of VD3 on UC progression, we carried out gene expression profile analysis in DSS-induced UC mouse colon tissues. In total, 1461 significant DEGs were identified, among which 798 were upregulated and 663 were downregulated (**Figure 3A**). Furthermore, we performed GO term and KEGG pathway analyses on the identified DEGs (**Figures 3B–D**). The gene expression levels of 16 enriched DEGs were depicted in a heatmap that were classified into vitamin B6 metabolism and pertussis signalling pathways, as determined by KEGG pathway analysis (**Figure 3C**). Among these 16 genes, VDR showed lower expression, but NLRP6, Caspase-1, PYD and CARD domain-containing (PYCARD) showed relatively higher expression in UC mouse colons (**Figure 3C**).

### VD_3_ suppressed NLRP6 inflammasome activation

**Figure 4:**
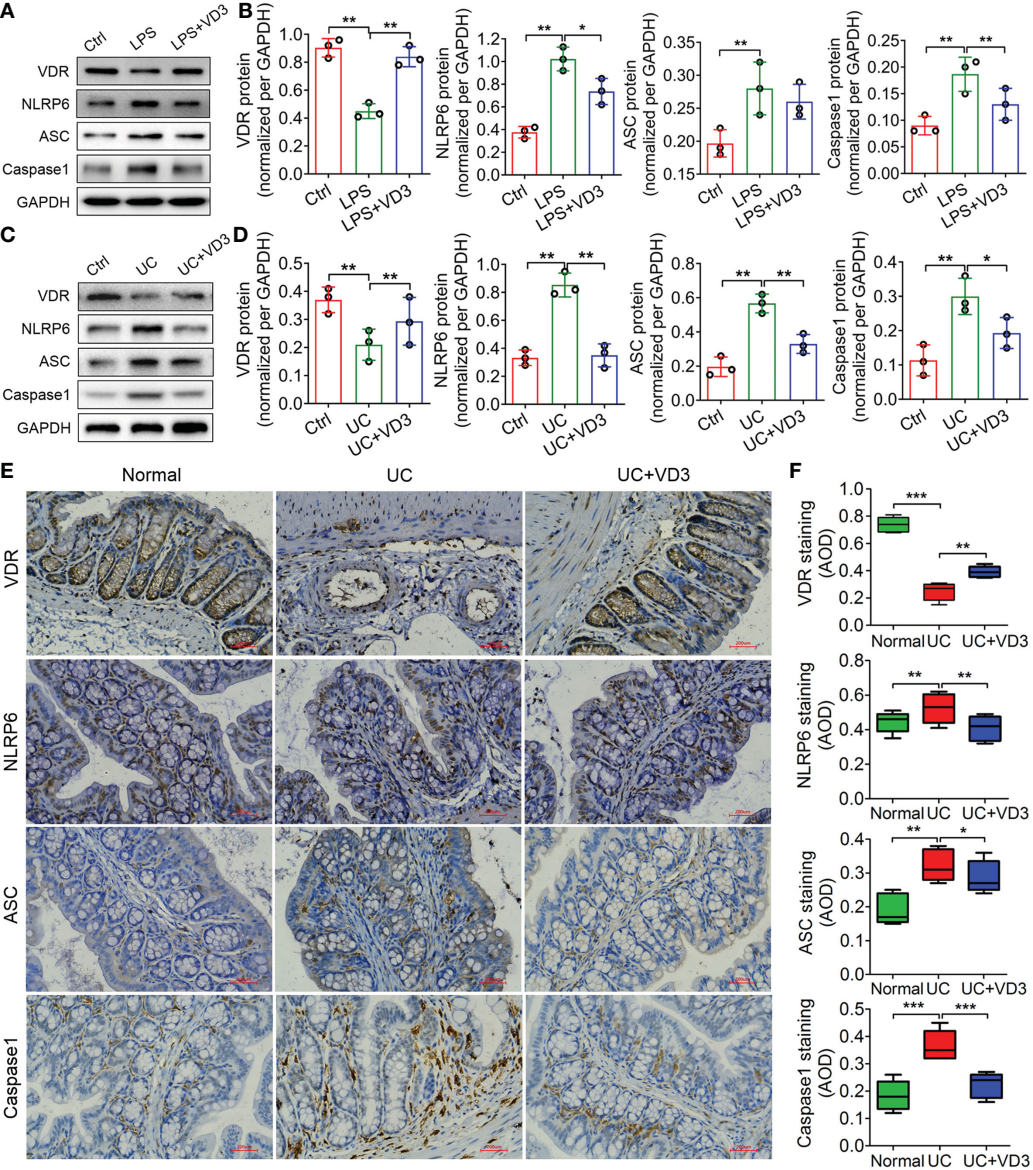
1,25 (OH)_2_D_3_ inhibits expression of the NLRP6, ASC and Caspase-1 inflammasomes in LPS-treated primary intestinal epithelial cells and DSS-induced UC mice. **(A)** Western blot analyses were performed to evaluate the expression of the VDR, NLRP6, ASC and Caspase-1 proteins in LPS-primed MIECs treated with VD_3_ for 3 h. **(C)** Western blot analyses were performed to evaluate the expression of the VDR, NLRP6, ASC and Caspase-1 proteins in UC mice treated with VD_3_. **(B)** and **(D)** The quantification of protein expressions in A and C. **(E)** Immunohistochemical analysis of VDR, NLRP6, ASC and Caspase-1 expression in colon sections from UC mice treated with VD_3_. **(F)** Quantitative analysis of the average optical density by immunohistochemistry. Scale bar represents 500 μm. The data are shown as the means ± SD; n ≥ 3, *P < 0.05, **P < 0.01, ***P < 0.001.

NLRP6 acts as an innate immune sensor that recruits ASC and Caspase-1 and forms an inflammasome mediating the release of the inflammatory cytokines IL-1β and IL-18 (30). Thus, we detected the expression of VDR and the main mediators of the NLRP6 inflammasome, NLRP6, ASC and Caspase-1, in UC mice and LPS-treated MIECs. The western blotting results showed that VDR protein expression decreased in both UC mice and LPS-treated MIECs compared to the controls (**P < *0.01*; **Figures 4A–D**). In contrast, the protein expression levels of NLRP6, ASC and Caspase-1 increased significantly in UC mice and LPS-treated MIECs (**P < *0.01*; **Figures 4A–D**). Moreover, the mRNA expression levels of these genes in UC mice and LPS-treated MIECs were consistent with the results of western blotting analysis (**P < *0.01*; **Supplemental Figure 1**). However, the expression of VDR was enhanced, but NLRP6 inflammasome gene expression was reduced by VD_3_ administration (*P < *0.05*, **P < *0.01*; **Figure 4**, **Supplemental Figure 1**). The immunohistochemistry results further confirmed these findings that VD_3_ significantly increased VDR expression but decreased NLRP6, ASC and Caspase-1 expression in UC mice (*P < *0.05*, **P < *0.01*, ***P < *0.001*; **Figures 4E, F**).

### VD_3_ transcriptionally regulates NLRP6 expression in mouse colons, and NLRP6 is a key player in the regulation of NLRP6 inflammasome activity in UC

**Figure 5:**
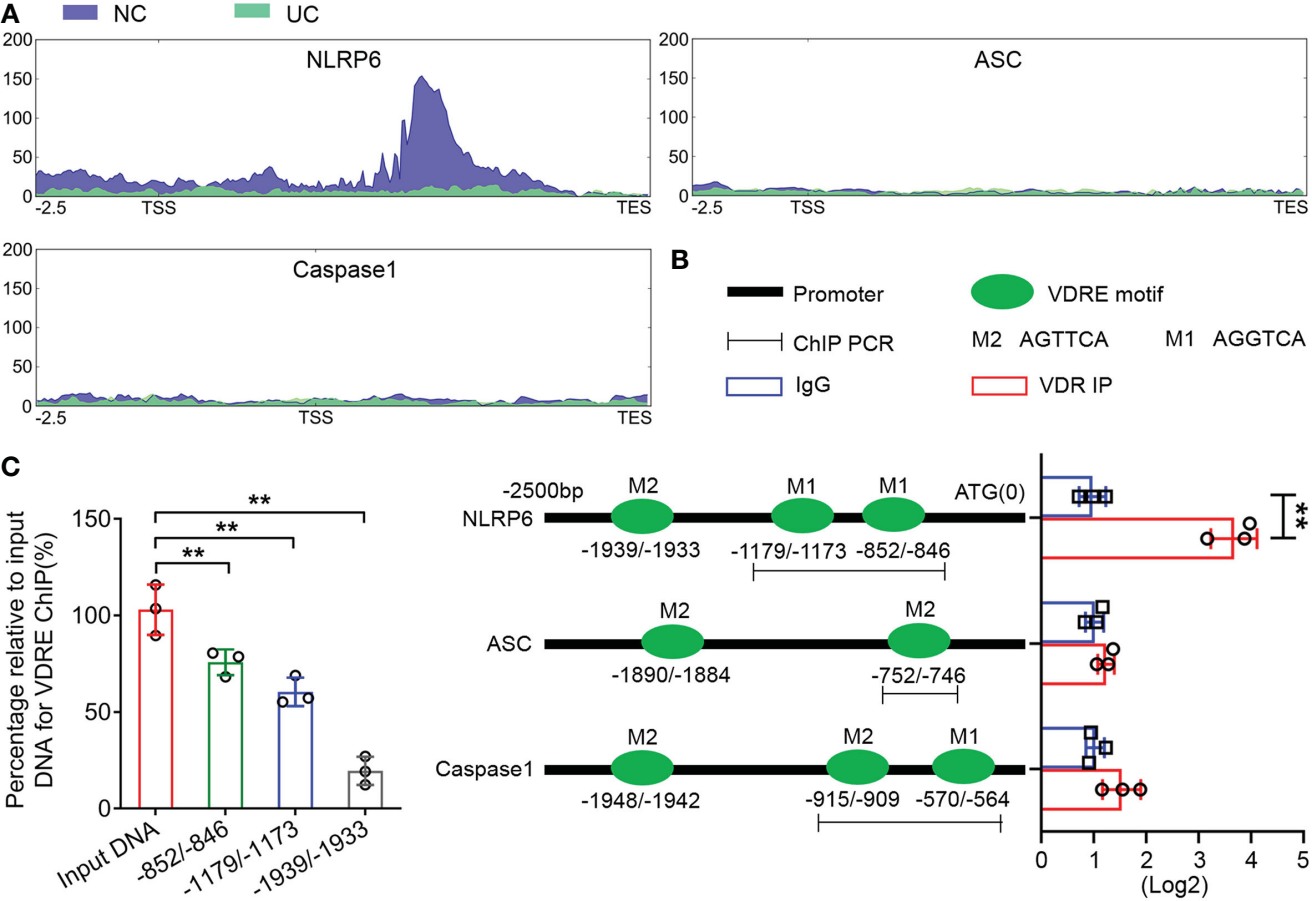
VDR downregulates NLRP6 expression by inhibiting the transcriptional activity of the NLRP6 promoter. **(A)** ATAC-seq enrichment from 2500 bp upstream of the TSSs throughout the whole ranges of the NLRP6, ASC and Caspase-1 genes in normal and UC tissues. **(B)** Presence of the AGTTCA and AGGTCA motifs in the promoters of NLRP6, ASC and Caspase-1 (left) and qChIP-PCR results (right) showing the binding of VDR to the promoter fragments containing the AGTTCA and AGGTCA motifs in the promoters of NLRP6, Caspase-1 and the AGTTCA motifs in the promoters of ASC, respectively. **(C)** The percentage relative to the input for the enriched fragments of the VDRE ChIP. The data are shown as the mean ± SD, n ≥ 3, **P < 0.01.

We performed ATAC-seq to map genome-wide chromatin accessibility to the colon tissues of UC mice. The results showed a peak in the NLRP6 promoter regions in the normal controls but not in the UC group (**Figure 5A**), which implied that NLRP6 was transcriptionally regulated during UC development. The potential binding sites in the NLRP6 promoter regions are shown in **Figure 5B**. In addition, the ChIP assay revealed that the DNA fragments containing -852/-846 and -1179/-1173 VDREs were enriched significantly compared to the IgG controls, while the fragment containing the -1939/-1933 VDRE was not enriched (**Figures 5B, C**). Meanwhile, the enriched DNA fragments were normalized to the input. Thus, the ATAC-seq and ChIP assays suggested that VDR transcriptionally bound to VDREs in mouse clone tissues.

**Figure 6:**
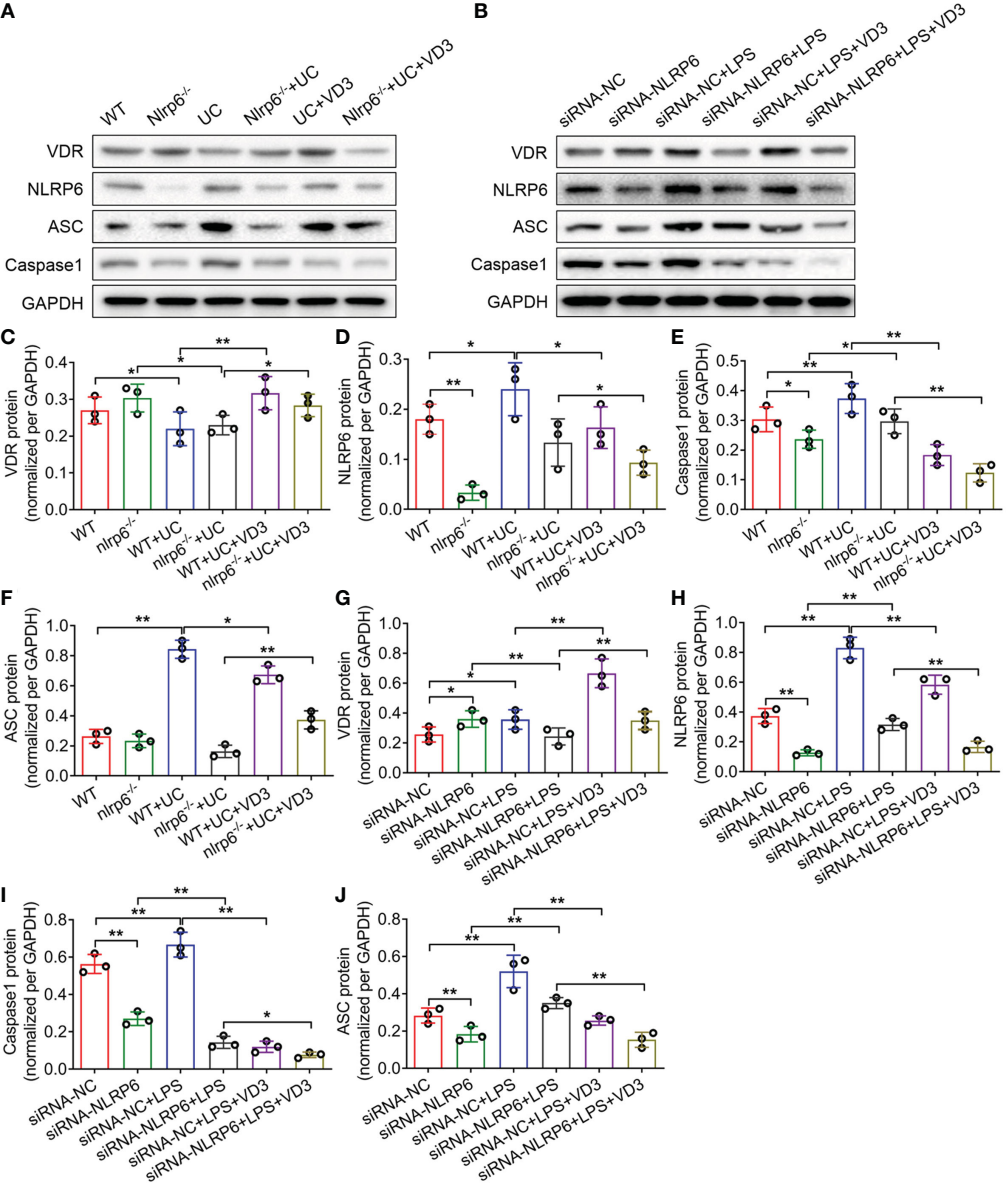
1,25(OH)_2_D_3_ promotes NLRP6 expression *in vivo* and *in vitro*. **(A)** Western blot analyses were performed to evaluate the expression of the VDR, NLRP6, ASC and Caspase-1 proteins in *Nlrp6* ^-/-^ mice and UC mice subsequently treated with VD_3_. **(B)** Western blot analyses were performed to evaluate the expression of the VDR, NLRP6, ASC and Caspase-1 proteins in NLRP6 siRNA MIECs that were then treated with LPS and incubated with VD_3_. **(C–J)** Quantitative analysis of the protein levels. The data are shown as the mean ± SD, n ≥ 3, *P < 0.05, **P < 0.01.

To identify the effect of NLRP6 on UC progression, we established *nlrp6* ^-/-^ mice and detected the expression levels of VDR, NLRP6, ASC and Caspase-1 in WT and *nlrp6* ^-/-^ mice treated with DSS and VD_3_. The western blotting results from the UC and UC + VD_3_ mice controlled by the WT mice were consistent with the above findings that VDR expression was reduced, but NLRP6, ASC and Caspase-1 expression levels were enhanced in UC mice (*P < *0.05*, **P < *0.01*; **Figure 6A, C–F**). However, VD_3_ significantly upregulated VDR but downregulated the expression of NLRP6 inflammasome genes (*P < *0.05*, **P < *0.01*; **Figures 6A, C–F**). The results from the LPS- and VD_3_-treated MIECs controlled by the siRNA-NC groups further confirmed these findings. However, in *nlrp6* ^-/-^ mice, the NLRP6 level was almost undetectable (**P < *0.01*; **Figures 6A, D**). DSS-induced UC increased NLRP6 levels in *nlrp6* ^-/-^ mice, but VD_3_ treatment decreased these levels (*P < *0.05*; **Figures 6A, D**). The levels of Caspase-1 and ASC were similar to those of NLRP6 in *nlrp6* ^-/-^ mice treated with UC and UC + VD_3_ (*P < *0.05*, **P < *0.01*; **Figures 6A, E, F**). Additionally, the results from siRNA-NLRP6 cells treated with LPS and LPS + VD_3_ further confirmed the results in *nlrp6* ^-/-^ mice (**Figures 6G**–**J**). Interestingly, VDR expression was increased in both *nlrp6* ^-/-^ mice compared to WT mice and in siRNA-NLRP6 cells compared to siRNA-NC controls (*P < *0.05*; **Figures 6A–C, G**). All these findings were confirmed by qPCR results (*P < *0.05*, **P < *0.01*; **Supplemental Figure 2**).

### VDR is negatively correlated with NLRP6 expression in the initiation and development of UC

**Figure 7:**
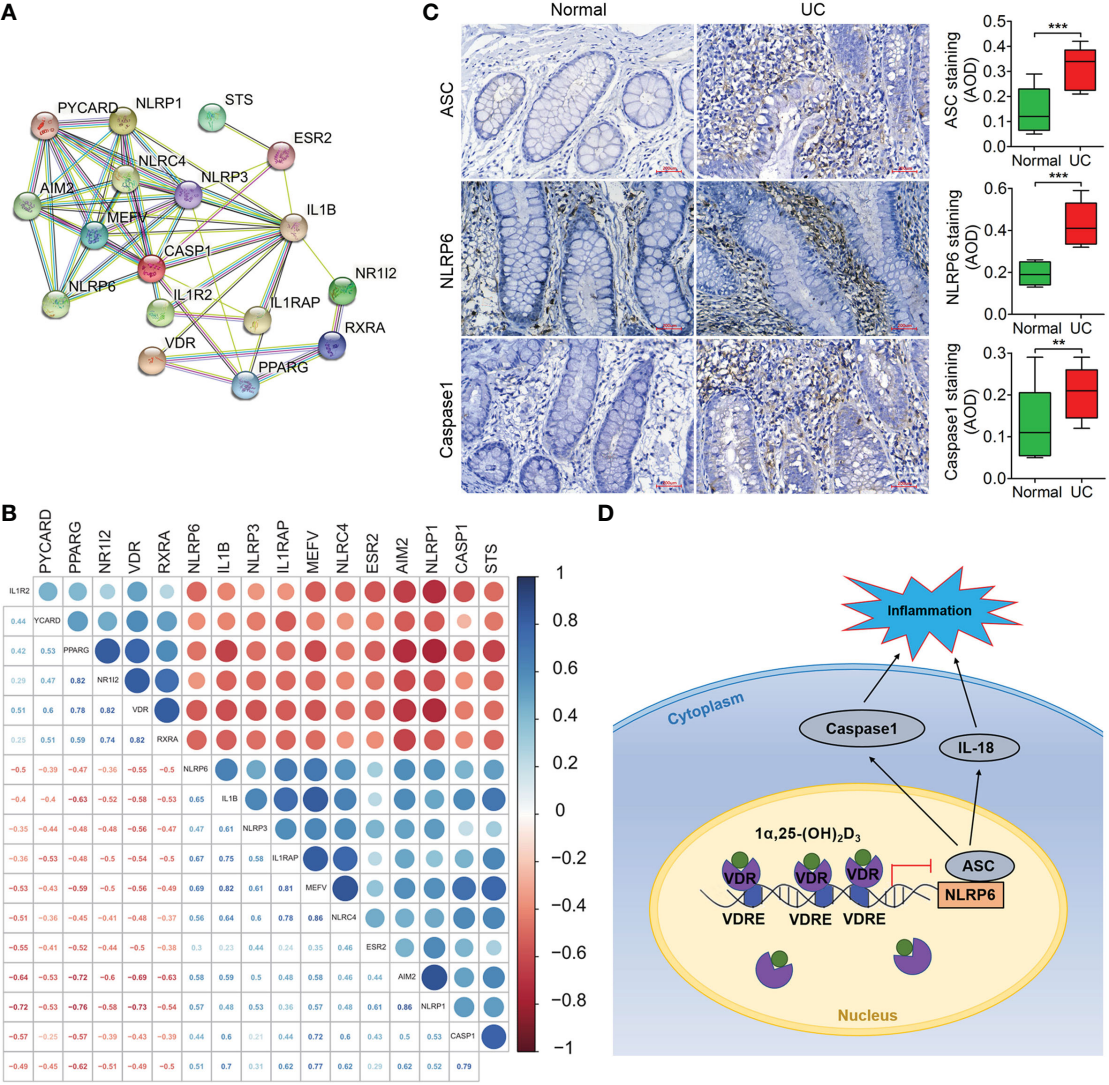
VDR expression is negatively correlated with NLRP6 expression and has the potential to be used for clinical prognosis prediction. **(A)** Protein–protein association network of the NLRP6-(Caspase-1)/IL-1β signalling pathway in STRING. VDR, NLRP6, ASC and Caspase-1 were selected as input. **(B)** Correlation of gene expression was analysed in GSE128682 ChiP data. The correlation coefficient ranges from −1 (red colour) to +1 (blue colour). The red region represents absolute negative correlations, afnd the blue region represents absolute positive correlations. Hclust, hierarchical clustering order. A value of 0.05 was chosen as the significance level. **(C)** Immunohistochemical analysis of ASC, NLRP6 and Caspase-1 expression in normal and UC tissue samples from representative patients. **(D)** Schematic illustration of VDR-NLRP6 signalling in MIECs. There are three VDREs in the promoter region of NLRP6. The transcription factor VDR transcriptionally represses NLRP6 by binding to the VDRE, reducing NLRP6 expression. The data are shown as the mean ± SD, n ≥ 3, *P < 0.05; **P < 0.01, ***P < 0.001.

The results from protein-protein association network analysis of the NLRP6-(Caspase-1)/IL-1β signalling pathway in STRING showed that VDR, NLRP6 and IL-1β were closely associated at the protein level (**Figure 7A**). Moreover, VDR expression was negatively correlated with NLRP6 and Caspase-1 expression (**Figure 7B**). Thus, we identified the expression of these NLRP6 inflammasome genes in UC patients, which showed that the protein expression levels of NLRP6, ASC and Caspase-1 were all increased compared to those in normal individuals in immunohistochemical and immunofluorescence analysis (***p < 0.001; **Figure 7C**, **Supplemental Figure 3**).

## Discussion

UC is a chronic inflammatory disease of the colon resulting in digestive disorders (2, 31). Although the incidence and prevalence have continued to increase worldwide in recent years, the current treatment methods for UC have not shown good clinical efficacy in most patients, who ultimately require colectomy, seriously affecting their quality of life (2). Factors associated with the onset and pathogenesis of UC have been uncovered recently, including genetic susceptibility, environmental factors, intestinal epithelial barrier dysfunction, and immune response dysregulation (32). However, many efforts are needed to enhance the understanding of UC development.

Vitamin D exerts various anti-inflammatory, antioxidant, immunomodulatory, and antifibrotic effects (33). Typically, patients with UC have low serum vitamin D levels, which are associated with complications such as low bone mineral density (34–37). The inverse association of vitamin D with IBD or UC disease has been confirmed recently, and vitamin D supplements have been shown to help relieve disease symptoms (33, 36, 38, 39). In this study, DSS-induced UC mice treated with VD_3_ showed a significant increase in body weight compared to UC mice, and VD_3_ administration successfully reduced the DAI and intestinal permeability rate in UC mice. Moreover, the results from colon sections revealed that VD_3_ supplements improved the integrity of the intestinal mucosal barrier. Thus, these results suggested that VD_3_ treatment attenuates pathological damage in UC mice, which confirmed previous findings. As the nuclear receptor of VD_3_, low expression of VDR and dysfunction of VD_3_-VDR signaling in IBD patients have been reported (40). In this study, the UC patients exhibited typical symptoms with obvious mucosal tissue defects and inflammatory cell infiltration. Meanwhile, the contents of the proinflammatory factors IL-1β and IL-18 increased significantly. However, the VDR protein level was reduced in UC patients. These results confirmed that VDR expression was inhibited in the intestinal tissue of patients with colitis. It is worth mentioning that the expression of the key enzymes in vitamin D metabolism releasing the active ingredient VD_3_ did not change in UC patients in this study, which indicated that UC development might not rely on the dysregulated metabolism of vitamin D, although its aberrances normally confer resistance to the protective effect of vitamin D in many diseases. Reduced VDR levels and the subsequent inactivation of VD_3_-VDR signaling contributed to UC progression.

According to the results from RNA-seq in DSS-induced UC mice, the expression levels of NLRP6 and Caspase-1 increased. NLRP6 is a recently defined inflammasome that plays key roles in regulating inflammation and intestinal homeostasis (41, 42). Although the association between NLRP6 expression and intestinal integrity has been confirmed, in some cases, NLRP6 expression was observed to be upregulated, while in others, it was downregulated (20). In this study, the expression levels of the NLRP6 inflammasome genes NLRP6, ASC and Caspase-1 were enhanced in UC patients and mice and in LPS-treated MIECs. Moreover, in UC mice and LPS-stimulated MIECs, the expression levels of these NLRP6 inflammasome genes were decreased, but VDR expression increased with VD_3_ treatment, which indicated that VD_3_-VDR signaling might be closely associated with NLRP6 inflammasome activation. The ATAC-seq and ChIP assays showed that VDR transcriptionally bound to VDRE in the NLRP6 promoter region, and the protein-protein association network indicated that VDR expression was negatively correlated with NLRP6 expression. Taken together, these results suggest that VDR transcriptionally represses NLRP6 expression and subsequently inhibits NLRP6 inflammasome activation. VDR-NRPL6 signaling might be responsible for the anti-inflammatory activity of VD_3_ in UC.

Interestingly, VDR expression increased in *nlrp6* ^-/-^ mice and siRNA-NLRP6 MIECs, which suggested that NLRP6 suppressed VDR expression in mouse colons. The feedback loop regulation between VDR and NLRP6 expression may benefit NLRP6 upregulation under UC conditions. In addition, the expression levels of ASC and Caspase-1 decreased with NLRP6 deficiency. Thus, NLRP6 might exert key roles in inflammasome assembly and activation. Recent research indicated that the disturbed interaction of NLRP6 with ASC prevented excessive inflammation (42).

In conclusion, we suggest that VDR might inhibit NLRP6 inflammasome activation by transcriptionally repressing NLRP6 expression in the animal intestine and that vitamin D exerts anti-inflammatory actions *via* VDR-NLRP6 signaling in UC development to maintain the stability of the intestinal mucosa (**Figure 7D**). Thus, this study clarified the molecular mechanism relating vitamin D protective roles in UC and accumulated evidence for the use of vitamin D in the clinical treatment of UC patients. Nonetheless, more investigations are still needed in the future, as the aetiological relationship between vitamin D and the onset of UC is far from clear.

## Data availability statement

The data presented in the study are deposited in the SRA repository, accession number PRJNA929335 (https://www.ncbi.nlm.nih.gov/bioproject/?term=PRJNA929335).

## Ethics statement

The studies involving human participants were reviewed and approved by the Ethics Committee of Xinjiang Medical University Affiliated Tumor Hospital. The patients/participants provided their written informed consent to participate in this study. The animal study was reviewed and approved by the Ethics Committee of Xinjiang Medical University Affiliated Tumor Hospital.

## Author contributions

HG performed the experiments, acquired the data and drafted the manuscript. HZ and JG revised the manuscript and analyzed and interpreted the data. ZZ and JL provided material and technological support. XL and HG conceived and designed the study, obtained funding and supervised the study. All authors contributed to the article and approved the submitted version.
