# Supplementary material for: Vitamin D3 alleviates inflammation in ulcerative colitis by activating the VDR-NLRP6 signaling pathway
Source: Front Immunol. 2023 Feb 8;14:1135930. doi: 10.3389/fimmu.2023.1135930 (PMC9944717; doi:10.3389/fimmu.2023.1135930)
Supplement: Supplementary file 1 [file DataSheet_1.docx]

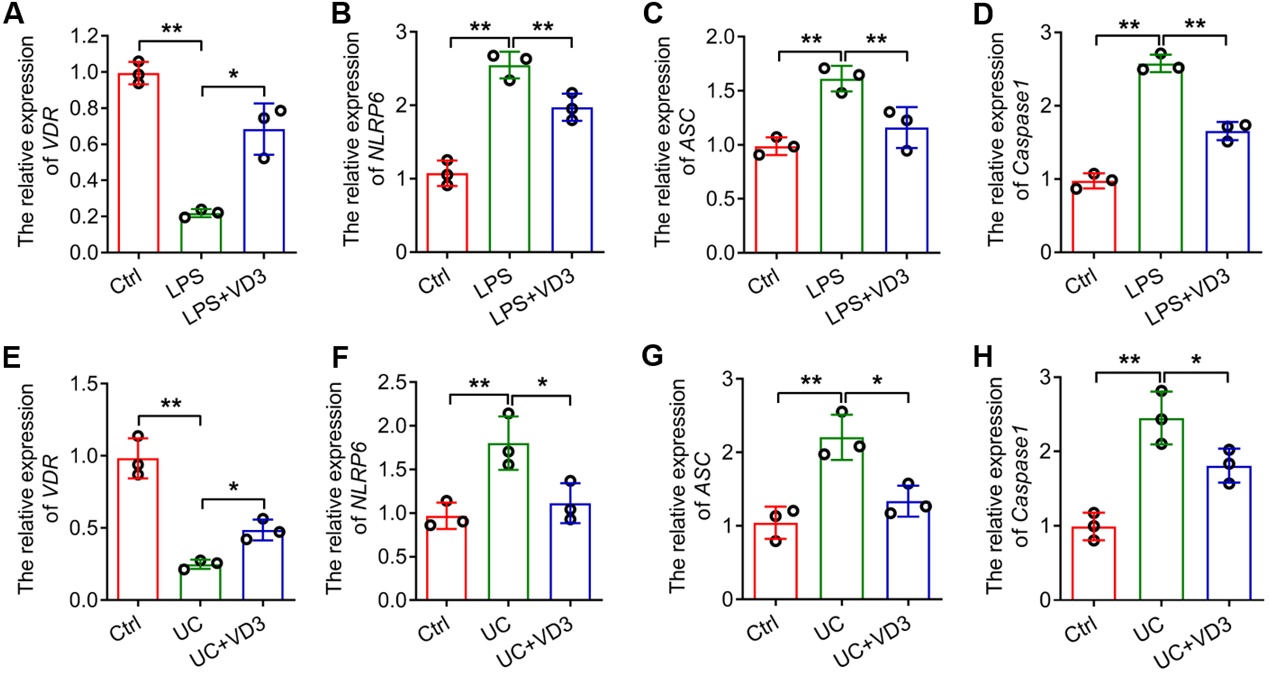
**Supplemental Figure 1** RT–PCR analysis of VDR, NLRP6, ASC, and Caspase-1 expression in LPS-primed MIECs treated with VD_3_ for 3 h (A-D) and in UC mice treated with VD_3_ (E-H). The data are shown as the means ± SD; n ≥ 3, **P < 0.05*, ***P < 0.01*.


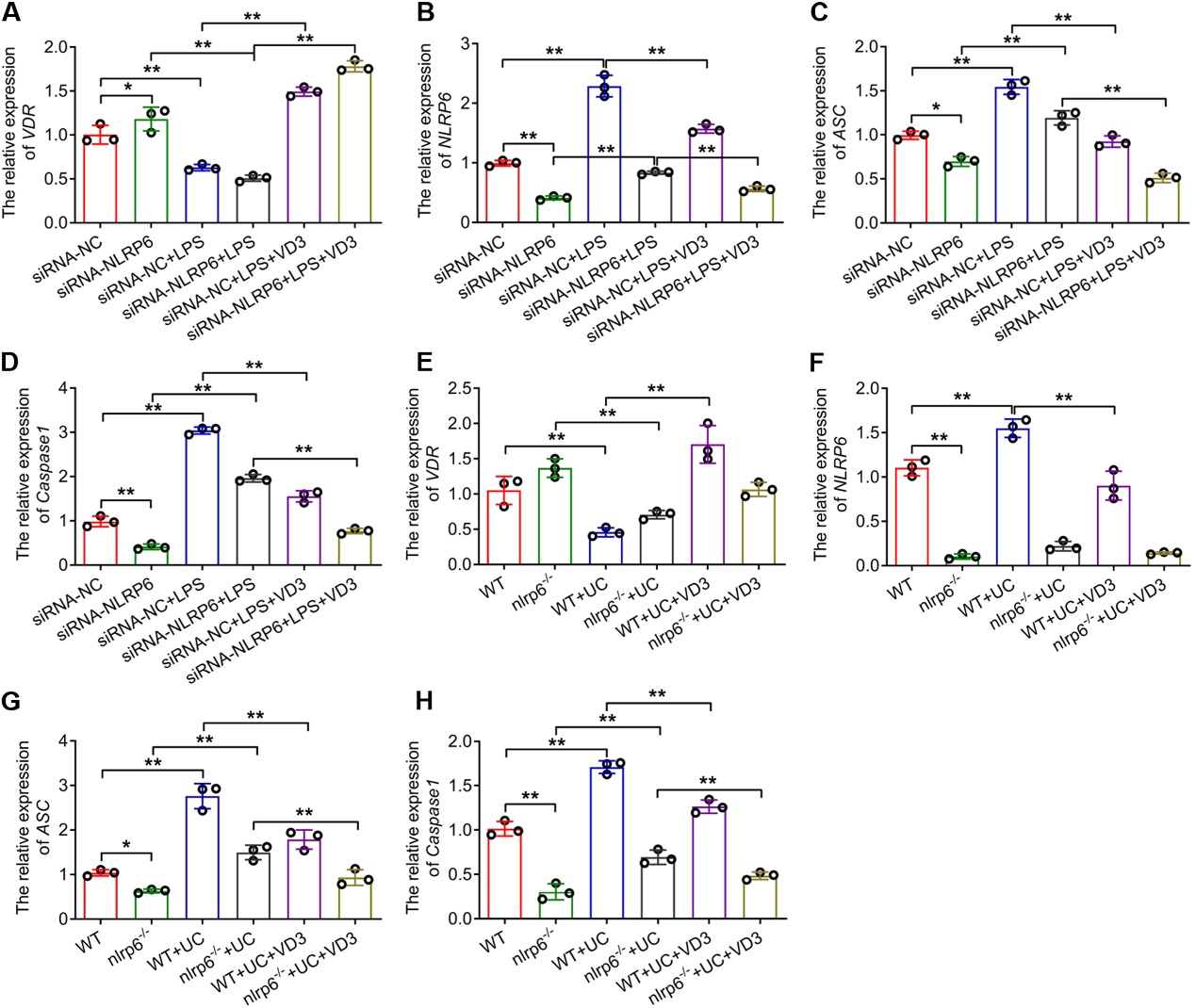


**Supplemental Figure 2** RT–PCR analysis of VDR, NLRP6, ASC, and Caspase-1 expression in MIECs transfected with NLRP6-siRNA from LPS-primed mice treated with VD_3_ for 3 h (A-D) and in *Nlrp6*^-/-^ mice induced by DSS, followed by incubation with VD_3_ (E-H). The data are shown as the means ± SD; n ≥ 3, **P < 0.05*, ***P < 0.01*.


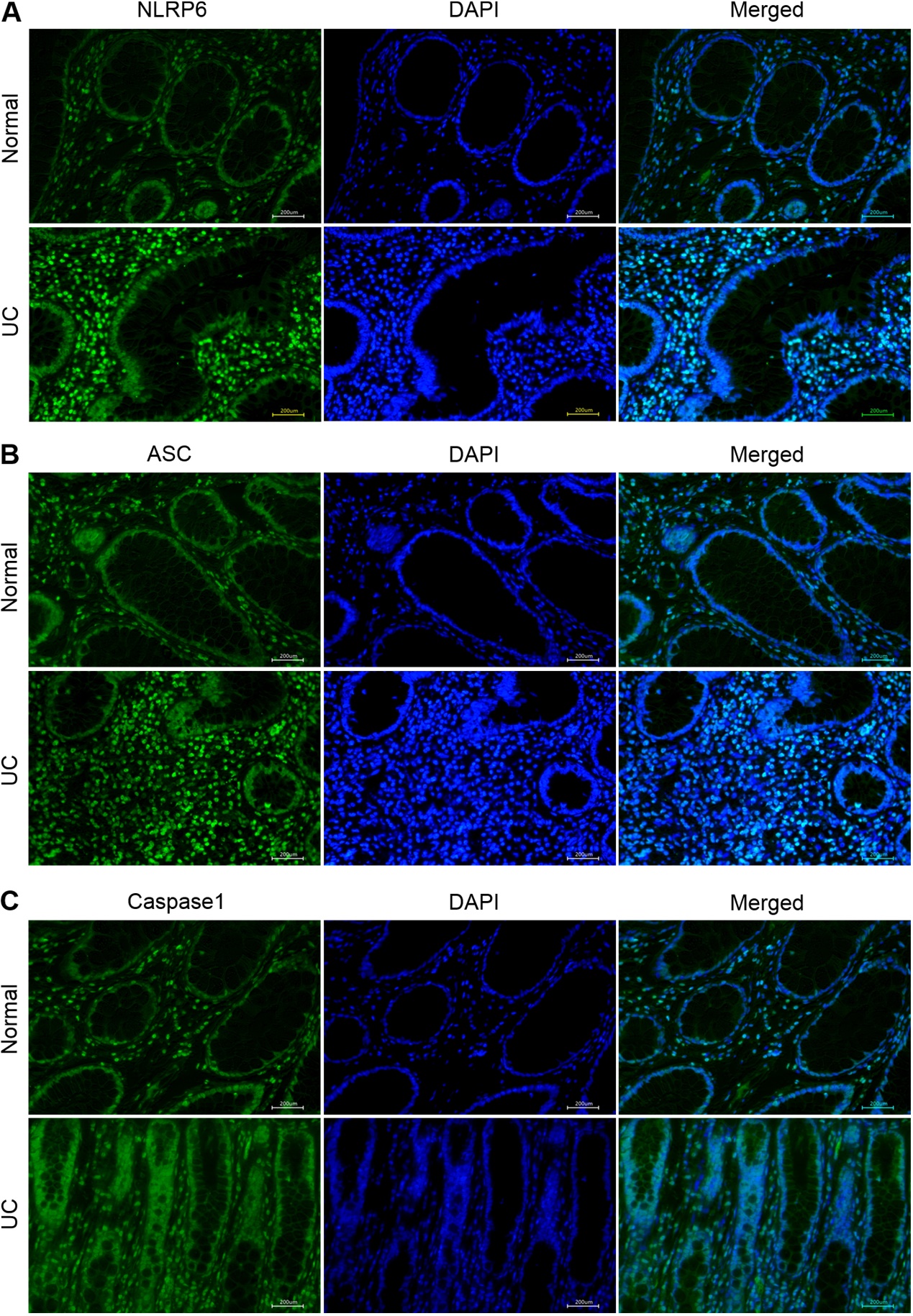


**Supplemental Figure 3** The expression of NLRP6 (A), ASC (B) and Caspase-1 (C) was measured by immunofluorescence in UC and normal tissue samples.
