# Supplementary material for: Vitamin D3 alleviates inflammation in ulcerative colitis by activating the VDR-NLRP6 signaling pathway
Source: Front Immunol. 2023 Feb 8;14:1135930. doi: 10.3389/fimmu.2023.1135930 (PMC9944717; doi:10.3389/fimmu.2023.1135930)
Supplement: Supplementary file 2 [file Table_1.docx]

**Table 1. The primer sequences in ChIP assay**

| Name | Forward Primer (5’ - 3’) | Reverse Primer (5’-3’） |
| --- | --- | --- |
| -852/-846 | CAGCTGTTATCAGCAATCCTGA | ACAGGATGTCTGGGACTTGGGA |
| -1179/-1173 | GAAATAGAGGCATAGAGGTTAA | GTAAAACACATACGTCTACATAA |
| -1939/-1933 | CCTCTATCACTATCACCTTGAA | GTTCTAACTCTGGAACCCACAG |
| NLRP6 | GAAATAGAGGCATAGAGGTTAA | GTTCTAACTCTGGAACCCACAG |
| ASC | CCCTTGGGAAGTAGAGTCAGGA | AGAATTTGATCCCAACACTGAT |
| Caspase1 | CTATTAATAAAATTATTTCACA | CAGGGTATCATTCTGTCGTGCAG |
